# Surgical options of chiasmatic hypothalamic glioma—a relevant part of therapy in an interdisciplinary approach for tumor control

**DOI:** 10.1007/s00381-024-06498-2

**Published:** 2024-06-25

**Authors:** Anna-Gila Karbe, David Gorodezki, Matthias Schulz, Anna Tietze, Arne Gruen, Pablo Hernáiz Driever, Martin U. Schuhmann, Ulrich-Wilhelm Thomale

**Affiliations:** 1 Pediatric Neurosurgery, Charité-Universitätsmedizin Berlin, Corporate Member of Freie Universität Berlin and Humboldt-Universität zu Berlin, Campus Virchow Klinikum, Augustenburger Platz 1, 13353 Berlin, Germany; 2 Department of Pediatric Oncology, University Children’s Hospital Tübingen, Tübingen, Germany; 3 Institute of Neuroradiology, Charité-Universitätsmedizin Berlin, Corporate Member of Freie Universität Berlin and Humboldt-Universität zu Berlin, Berlin, Germany; 4 Department for Radiation Oncology and Radiotherapy, Charité-Universitätsmedizin Berlin, Corporate Member of Freie Universität Berlin and Humboldt-Universität zu Berlin, Berlin, Germany; 5 Department of Pediatric Oncology and Hematology; German HIT-LOGGIC-Registry for Low Grade Glioma in Children and Adolescents, Charité-Universitätsmedizin Berlin, Corporate Member of Freie Universität Berlin and Humboldt-Universität zu Berlin, Berlin, Germany; 6 Section of Pediatric Neurosurgery, Department of Neurosurgery, University Hospital of Tübingen, Tübingen, Germany

**Keywords:** Low-grade glioma, Optic pathway glioma, Chiasmatic hypothalamic glioma, Microsurgery, Neuroendoscopy, Hydrocephalus, Cysts

## Abstract

**Objective:**

The extent of resection of pediatric low-grade glioma mostly improves progression-free survival. In chiasmatic hypothalamic glioma (CHG), complete resections are limited due to the relevantly high risk of associated neurological and endocrinological deficits. Still, surgery might have its role in the framework of a multidisciplinary team (MDT) approach. We report our retrospective experience from two centers on surgical options and their impact on long-term outcomes.

**Methods:**

Medical records of surgically treated pediatric CHG patients between 2004 and 2022 were analyzed. Patient characteristics, surgical interventions, histology, and non-surgical therapy were retrieved together with outcome measures such as visual acuity, endocrine function, and survival.

**Results:**

A total of 63 patients (33 female, NF-1, *n* = 8) were included. Age at first diagnosis was 4.6 years (range 0.2–16.9) and cohort follow-up was 108 ± 72 months. Twenty patients were surgically treated with a biopsy and 43 patients with debulking at a median age of 6.5 years (range 0.16–16.9). Patients received a median of 2 tumor surgeries (range 1–5). Cyst drainage was accomplished in 15 patients, and 27 patients had ventriculoperitoneal shunt implantation. Non-surgical therapy was given in 69.8%. At the end of follow-up, 74.6% of patients had stable disease. The cohort had a median Karnofsky score of 90 (range 0–100). Four patients died. Hormone substitution was necessary in 30.2%, and visual acuity was impaired in 66% of patients.

**Conclusion:**

Pediatric CHG is a chronic disease due to overall high survival with multiple progressions. Surgical therapy remains a key treatment option offering biopsy, limited tumor-debulking, cyst fenestration, and hydrocephalus management in the framework of MDT decision-making. Team experience contributes to reducing possible deficits in this challenging cohort.

## Introduction

Low-grade glioma (LGG) represents the most frequent childhood central nervous system tumor. Surgery is considered the mainstay of therapy, with a realistic chance for oncological cure in case of gross total resection. However, certain tumor locations and entities represent no indication for complete resection because of intrinsic growth patterns and eloquent tumor location associated with a relevant high risk of functional deterioration following complete surgery. Supratentorial midline location tumors often involve the optic pathway besides the hypothalamus. They are anatomically characterized by the Dodge classification dividing optic nerve and chiasmatic and optic tract involvement [1]. In the HIT LGG 1996 study with 1031 pediatric LGG (pLGG) patients, optic pathway glioma (OPG) accounted for 22.4% of patients, while chiasmatic hypothalamic glioma (CHG) accounted for the vast majority (90%) of OPG [2].

The core tumor volume of CHG is typically located in the suprasellar region, and magnetic resonance (MR) imaging is the standard diagnostic tool to determine the pattern and extent of growth. Tumors often show heterogenous lesion patterns in MRI including contrast- and non-contrast-enhanced tumor areas. T2-weighted images reveal different outlines of intensity signals within the tumor, which sometimes include cystic parts. The typical imaging characteristics well distinguish CHG from other suprasellar tumor lesions in children [3]. The subtype (cyst, solid part intensity pattern) of the lesion on imaging which is growing indicates the dynamics of tumor genesis [4]. Hydrocephalus typically occurs when the tumor occupies major parts of the third ventricle, leading to bilateral foramen of Monro or Sylvian aqueduct occlusion.

Despite the eloquence of the location biopsy, partial tumor debulking, cyst drainage, and hydrocephalus treatment remain relevant surgical options in the management of these tumors [5]. The radicality of resection is limited by the high risk of causing quality-of-life-impairing conditions such as diencephalic syndrome, blindness, hormone deficiencies, and cognitive and motor disabilities. Chemotherapy, radiation therapy, and even observational strategy are legitimate options for management [6]. Targeted therapies addressing the MAP kinase pathway have been introduced addressing patients without surgical options or failed treatment of standard LGG chemotherapy [7–9]. Given the variety of presentation patterns and therapeutic options, often also depending on age or underlying genetic syndromes like NF1, treatment decisions for the individual patient must be made by an experienced team through multidisciplinary case discussions to identify the best possible treatment at any given stage of disease development [5].

The overall survival rate of patients is relatively high while progression-free survival is low, defining CHG as a chronic disease. The risk of tumor progression depends on the age at diagnosis and at the start of therapy. Patients with tumor histology of pilocytic astrocytoma fare better than those with non-pilocytic histology. Diencephalic syndrome at presentation as well as tumor dissemination at diagnosis further impairs overall and progression-free survival. A positive genetic status of neurofibromatosis type 1 (NF1) improves overall, event, and progression-free survival [2, 10].

The aim of managing patients suffering from this chronic tumor disease is to preserve the quality of life by optimizing tumor control with the lowest rate of treatment associated neurotoxicity. The role of surgery has changed since gross total resection was the standard of treatment for low-grade glioma up until the 1990s in Germany without taking tumor location into account. After reports on catastrophic postoperative courses, surgical interventions were almost avoided since the 1990s and the beginning of the new millennium. Lately, the role of surgery has become increasingly more nuanced. While treatment of hydrocephalus and mass-occupying tumor cysts are clear surgical indications, the role of tumor resection was defined by identifying exophytic tumor parts with relevant mass effect, a high signal in T2-weighted imaging, and safe surgical strategies [11].

Our current study retrospectively reviews a patient cohort with chiasmatic-hypothalamic glioma in children and adolescents in two German pediatric neurosurgery centers. We analyzed the type and number of surgical interventions. Eventually, we evaluated whether histology and non-surgical therapy further defined outcome measures such as visual acuity, endocrine function, and survival.

## Methods

The study included patients under the age of 18 years presenting with chiasmatic hypothalamic glioma surgically treated in the departments of pediatric neurosurgery of Charité-Universitätsmedizin Berlin as well as Universitätsklinikum Tübingen, Germany. Patients were collected from in-house patient databases as diagnosed and treated at least with one tumor surgical intervention between 2004 and 2022 in a retrospective analysis. Possible additional previous tumor surgeries were also documented. The ethics commission approval was given by the Ethics Committee of the Charité for this study (EA2/005/24).

### Data collection

Patients’ characteristics included sex, age at initial diagnosis and at first tumor surgery, NF1 status, possible dissemination, tumor localization, and histological diagnosis. Surgical treatment included time, type, and amount of tumor surgeries including biopsies and debulking surgeries. Cyst fenestrations and hydrocephalus treatment such as endoscopic third ventriculostomy and ventriculoperitoneal shunt placements were recorded. Non-surgical treatment regimen was recorded, i.e., chemotherapy, targeted therapy, and radiation therapy. At the end of the follow-up, visual acuity, hormonal deficits, auxological data, Karnofsky score, and tumor status were recorded.

For visual and hormonal function, any kind of impairment was rated as present. Age-related BMI evaluation was used to evaluate the presence of obesity. Tumor status was categorized as stable (SD), progressive disease (PD), complete remission (CR) of disease or death.

### Tumor surgery

Aims of tumor surgery were defined at multidisciplinary team conferences that included all relevant disciplines such as Pediatric Neurooncology, Radiation Oncology, Pediatric Neurosurgery, Pediatric Neuroradiology, Neuropathology, and Pediatric Neurology. Possible treatment options were weighted against each other in terms of possible neurotoxicity at any given time of the disease course as well as clinical signs and symptoms reported by patients.

The options for surgical tumor interventions were first to decide between tumor biopsy (diagnostic) or tumor debulking (therapeutic). When the goal was purely diagnostic, a biopsy was performed to define diagnosis including molecular genetic subclassification or in case of tumor progression to exclude malignant transformation. Biopsies were performed microsurgically, endoscopically, or stereotactically depending on the surgeon’s preference and accessibility of the tumor.

**Fig. 1:**
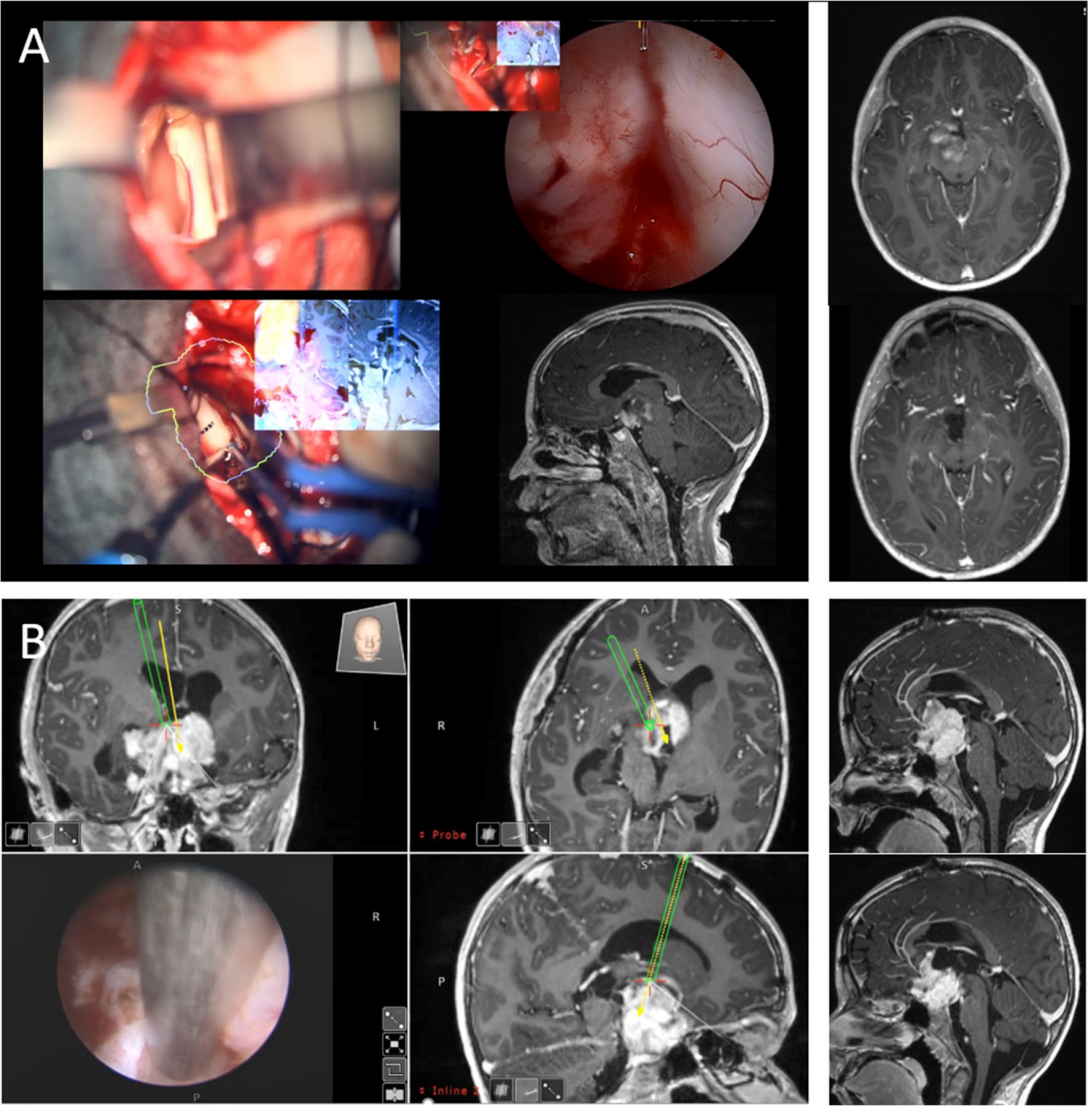
**A** Surgical microscopic view into a third ventricular hypothalamic tumor part, which was excised via a trans-callosal, trans-foramen-of-Monro approach using augmented reality assisted neuronavigation assisted by intraoperative endoscopic inspection for resection cavity in deep areas for resection control. **B** Pure navigated endoscopic debulking technique of the third ventricular tumor part to free the CSF pathways and to avoid hydrocephalus progression and shunting. Chemotherapy was started after debulking surgery in case B.

For therapeutic tumor debulking, T2-weighted imaging was predominantly used to define the exophytic part of the tumor, which typically shows a high-intensity signal with high tissue water content. This tumor portion would be judged to be a target for debulking if a significant mass effect was present or if CSF circulation was blocked by this part of the tumor and if the tumor part was safely accessible by any selected approach. During surgery, usually, neuronavigation as well as intraoperative ultrasound or magnetic resonance imaging was applied. The goal was to address primarily the softer type of tissue with higher water content in T2 imaging and greyish color intraoperatively with gel-type consistency which can be resected safely by an ultrasonic aspirator. More firm tumor tissue, often of yellowish color, was considered to be rather of an intrinsic nature and was kept mostly untouched during the intervention. Tumor debulking surgery was mostly performed by microsurgical technique. Typical approaches were the transcallosal approach for third ventricular tumor parts (Fig. 1A), subfrontal supraorbital approach for anterior extended tumor parts, and rarely pterional approaches when the tumor extended laterally. In the more recent series in the Berlin cohort, the endoscopic technique for tumor debulking was applied [12]. This technique utilizes a single borehole and the navigated endoscopic approach using the MINOP INVENT (Braun-Aesculap, Germany) together with the Endoscopic Neurosurgical Pen (ENP, Söring, Germany) as ultrasound aspirator applied within the working channel of the neuroendoscope (Fig. 1B). Thereby, the aim of tumor debulking included reducing the mass effect of the tumor to gain time to postpone any kind of non-surgical tumor therapy or to resect the third ventricular part of the tumor to free the CSF pathway and avoid the implantation of a shunt system.

### Cyst and hydrocephalus treatment

In some individuals, tumors develop cystic components with their own growth dynamic leading to compression of unaffected brain tissue or blocking of CSF pathways. In some conditions, cysts might occur together with growing solid exophytic tumor parts. In this situation, cysts might be helpful during surgery to select a safer approach, to gain significant volume reduction for better accessibility of the solid tumor parts, or to better delineate the tumor margins during surgery. Some cysts may be addressed by surgery isolated from the solid tumor mass if only the cyst was growing but the solid tumor volume remained stable. Hereby, the aim is to establish a connection between the cyst and regular CSF spaces. This was achieved by cyst fenestration, mostly performed by navigated neuroendoscopy or by implantation of a cyst stent connected to the ventricular system. The stent was usually designed with a limited amount of perforation holes at the tip and applying additional perforation holes more proximally by a 2-mm rongeur at the length defined by preoperative image planning [13].

Hydrocephalus occurs through different preconditions [14]. A non-communicating hydrocephalus is present due to direct occlusion of the major CSF pathways by a tumor and/or cystic mass, predominantly the third ventricle. The surgical aim in this circumstance was to free the CSF pathway by directly approaching the mass lesion e.g. in the third ventricle itself. If this was not applicable, an alternative approach was applied. One option is an endoscopic third ventriculocisternostomy (ETV), especially if a pressure gradient between internal and external CSF spaces was present and occlusion of the Sylvian aqueduct or the outlets of the 4th ventricle were seen. This ETV was often secured with a catheter stent with perforation holes in the prepontine cistern as well as in the ventricle fixated with a reservoir at the bur hole level as described previously [15] since the floor of the third ventricle was often occupied by tumor tissue. Otherwise, a ventriculoperitoneal shunt (VPS) was implanted, which is also the common approach for hydrocephalic conditions in disseminated tumor disease.

### Statistical analysis

Data were summarized using Excel (MS Office, Microsoft, USA) data sheets, and mean ± standard deviation was calculated if values were equivalent to the median (given normal distribution); otherwise, the median was given in combination with the range, accordingly. Frequencies were defined by absolute and relative numbers.

## Results

A total of 63 patients met the inclusion criteria. Twenty patients were collected from the Tübingen series and 43 patients from the Berlin cohort (33 female, 30 male, Table 1). Median age at first diagnosis was 4.6 years (range 0.2–16.9). NF-1 status was verified in 8 patients (12.7%). All included tumors were defined as chiasmatic hypothalamic tumors. The predominant tumor location was the anterior chiasm in 17 patients, the chiasm in 34 cases, bilateral tracts in 3, and one unilateral tract in 9 children. The mean follow-up time was 108 ± 72 months (range 16–326). Two children developed tumor dissemination during the course of the disease.

**Table 1:** Patient characteristics (values are given as absolute numbers (percentage rate) and median and range if not addressed otherwise)

*n*	63
Time period	2004–2022
Sex distribution	♀ 33; ♂ 30
Age at diagnosis	4.6 years (range 0.17–16.9)
Age < 1 yr at diagnosis	*n* = 12 (19%)
NF-1	*n* = 8 (12.7%)
Follow-up time	103 months (range 16–326)
Age at tumor surgery	6.5 years (range 0.17–16.9)
# of tumor surgeries	2/patient (range 1–5)
Type of 1st surgery	*n* = 43 debulking (68%)
	*n* = 20 biopsy (32%)
Neuropathology	*n* = 53 pilocytic astrocytoma WHO I° (84%)
	*n* = 3 pilocystic astrocytoma subtype WHO I° (4.8%)
	*n* = 1 diffuse astrocytoma WHO II°
	*n* = 1 ganglioglioma WHO I°
	*n* = 1 RGNT WHO I°
	*n* = 1 oligodendroglioma WHO II°
	*n* = 1 astroglial tumor with high proliferation rate
	*n* = 1 chondrosarkoma (sec. malignancy)
	*n* = 1 not specified
# of medical therapies	1/patient (range 0–5)
Chemotherapy rate	*n* = 42 (66.7%)
Targeted therapy rate	*n* = 10 (15.9%)
Radiation therapy rate	*n* = 9 (14.3%)
Hydrocephalus rate	*n* = 30 (47.6%)
Shunt rate	*n* = 27 (42.9%)
ETV rate	*n* = 3 (4.8%)
Cyst fenestration rate	*n* = 16 (25.3%)
Visual impairment	*n* = 42 (66.7%)
Hormone deficiency	*n* = 19 (30.2%)
Weight	*n* = 2 (3.2%) underweight
	*n* = 35 (55.6%) normal weight
	*n* = 25 (39.7%) obesity
Karnofsky score	90 (range 10–100)
Karnofsky ≥ 70	*n* = 53/63 (84.1%)
Tumor status	*n* = 47 (74.6%) SD
	*n* = 12 (19%) PD
	*n* = 4 (6.3%) death

### Tumor surgeries

**Fig. 2:**
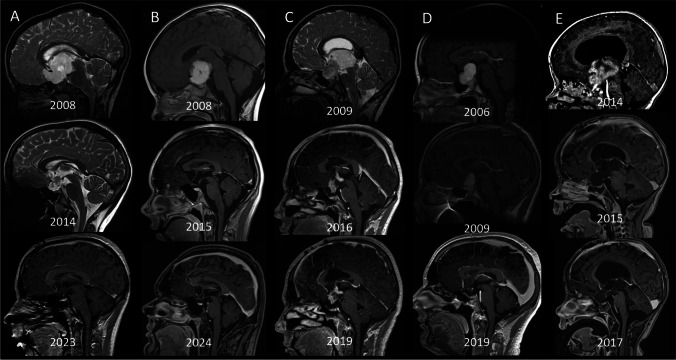
Case examples with long-term follow-up representing the multimodal treatment approach in CHG including relevant surgical strategies. **A** A 4-year-old boy presented with visual impairment had a biopsy. Neuropathology revealed pilocytic astrocytoma subtype pilomyxoid WHO I°. Standard LGG chemotherapy regimen (carboplatin/VCR) resulted in relevant decrease of the tumor mass although other systemic treatment lines needed to follow. Visual acuity at follow-up: right 0; left 1.0. **B** A 4-year-old boy presenting with nystagmus showed relevant tumor mass with exophytic tumor components. Staged debulking surgeries were scheduled and one chemotherapy was administered finally reaching stable disease. **C** A 9-year-old boy presented with nausea and vomiting. MRI revealed a hydrocephalus and a CHG. An interhemispheric trans-callosal resection of T2 hyperintense, 3rd ventricular part was performed twice during disease, eventually reaching stable disease. **D** A 12-year-old boy with visual impairment was initially treated through a subfrontal lateral approach. After developing further progression at the age of 15 years, another debulking was achieved followed by radiation. Visual acuity at follow-up: right 0.4; left 1.0; visual field assessment revealed incomplete hemianopia; **E** A 4-year-old boy suffering from headache and irritability had trans-callosal debulking surgery. Despite the freed 3rd ventricle, ongoing hydrocephalus required VP shunt implantation and MRI showed secondary tumor dissemination. Despite multiple lines of chemotherapy-refractory tumor progression was observed, and the patient died at the age of 7 years

The median age of initial tumor surgery was 6.5 years (range 0.16–16.9). The vast majority of tumor surgeries were performed near initial diagnosis (< 3 months) (*n* = 38). Eight children received surgery within 1 year, 7 patients within 5 years, and the remaining 9 patients were operated on more than 5 years after initial diagnosis. The initial approach was biopsy in 20 patients and tumor debulking in 43 children. The median number of tumor surgeries during follow-up was 2 surgeries per patient (range 1–5) including previous surgeries ex domo. Subsequent follow-up tumor surgery was performed in 32 patients after a median time interval of 29.5 months (range 0–309). In this group, three patients received a biopsy, and 29 children received another tumor debulking surgery. The third follow-up surgery took place in 12 children with a median time interval to the previous surgery of 13.5 months (range 1–142). Examples of the course of surgical treatments are presented in Fig. 2.

### Neuropathology

Since molecular genetic diagnosis was established during the course of the study period on a routine basis, the related data remain incomplete for the whole cohort. Thus, classic histological diagnoses according to the WHO classification at the given time point were as follows. The vast majority of patients showed a pilocytic astrocytoma (PA) WHO 1° (*n* = 53). Three PA subtype pilomyxoid WHO 1° were diagnosed together with one diffuse astroglial tumor WHO 2°, one rosette-forming glioneuronal tumor (RGNT) WHO 1°, one ganglioglioma WHO 1°, one oligodendroglioma WHO 2°, and one chondrosarcoma (after initial diagnosis of a PA). One patient showed an astroglial tumor with increased proliferative activity not being specified in further detail, and one child received a stereotactic biopsy that did not define the precise tumor diagnosis.

### Non-surgical treatment

Systemic anti-tumor treatment was applied in 42 patients (66.7%) and included carboplatin and vincristine w/o etoposide, vinblastine monotherapy, and the combination of thioguanine, procarbazine, CCNU, and vincristine LGG chemotherapy protocols (vinblastine alone, LGG 1996, SIOP, LGG 2004, TPCV). The median number of medical treatments applied was 1 (range 0–5). Twenty-four children received at least one type of systemic treatment, while 17 patients received 2 or more systemic treatment lines (two lines in 13, 4 lines in 2, and 5 lines in 2 children). Fourteen patients received neo-adjuvant systemic treatment, while 33 patients received adjuvant systemic treatment. Ten patients received targeted therapy, i.e., dabrafenib, trametinib, or biologicals such as bevacizumab. Four patients eventually received a high-grade chemotherapy regimen due to transformation to aggressive tumor growth dynamics.

Radiation therapy was applied in 9 children. One patient received radiation before surgery while the remainder was irradiated after surgery. Two received proton beam therapy, 6 received focal hyperfractionated radiation therapy, and 1 patient received stereotactic seed implantation.

### Cyst and hydrocephalus treatment

Isolated surgical cyst drainages were accomplished in 15 patients. The total amount of cyst fenestrations were 29 (median. 1/patient; range, 1–8; *n* = 9—1 fenestration; *n* = 3—2 fenestrations; *n* = 3— ≥ 3 fenestrations). Most cyst fenestrations were performed endoscopically (*n* = 16).

A total of 30 patients received dedicated surgical hydrocephalus treatment during the disease course, while 27 patients had a ventriculoperitoneal shunt implantation. The median number of shunt surgeries during follow-up was 1 (range 1–8), while 10 patients received 2 and 7 patients received even more shunt surgeries (3 surgeries, *n* = 3; ≥ 4 surgeries, *n* = 4).

### Outcome parameters

Outcome variables were evaluated at the date closest to the end of follow-up. Impairment in visual acuity was detected in 42 patients (66.7%). Amaurosis on one eye was observed in 25% of patients (*n* = 16) and on both sides in 15.9% of patients (*n* = 10).

Hormone deficiencies were documented in 19 individuals (30.1%). Weight data were missing preoperatively in 11 patients and at the end of follow-up in one. Looking at changes before and after treatment, 35 patients showed normal weight measures at both time points. The amount of low-weighed children was higher before compared to the end of follow-up (*n* = 13 vs. *n* = 2), while the number of overweight children increased from *n* = 4 to *n* = 25.

Karnofsky performance score reached the vast majority values of ≥ 70 (*n* = 53; 84.1%). Two patients were slightly disabled with a performance rating of 60 (*n* = 1) and 50 (*n* = 1), and 4 patients were disabled (KPS of 40). Four patients (6.5%) of this cohort died at the mean age of 8.4 ± 6.1 years with a median of 3.7 years after diagnosis (range 1.3–12.5).

Tumor status at the end of follow-up was stable disease (SD) in 47 patients (74.6%) at a mean age of 15 ± 6.6 years and progressive disease (PD) in 12 children (19%) at an age of 13.4 ± 5.6 years.

## Discussion

The current study analyzed the disease course of 63 patients who had any surgical treatment as part of pediatric chiasmatic hypothalamic glioma management. We observed that surgical therapy was an integral part of management to achieve safe tumor control as far as possible. During a mean follow-up period of 108 ± 72 months, a mean number of 1.85 ± 1 tumor surgeries per patient and 1.1 ± 1 medical anti-tumoral therapies per patient were applied while 14.3% of patients received radiation therapy. In addition, 42.9% of patients needed a VP shunt with a mean number of 2.1 ± 1.8 shunt surgeries, and 43.1% of patients had a mean number of 1.9 ± 1.8 isolated cyst surgeries during follow-up. These multiple and diverse steps of treatment underscore the importance of a multidisciplinary team approach when following patients and making individual decisions for whether an intervention is necessary and if so which is the best option. Communication with patients and parents needs to stress right from the very beginning that a CHG diagnosis represents a chronic disease most likely necessitating multiple steps of treatment in years to come.

The HIT 1996 study report focused on the management of LGG patients including the CHG cohort in [10] and did not focus on surgical therapy since the grade of resection was exclusively mentioned alongside other patient characteristics such as age, sex, NF status, and tumor localization. Nevertheless, 72.2% of the patients received some kind of tumor surgery, and almost half (33.8%) received biopsy alone during a median follow-up time of 42.1 months. In five patients of that cohort, a complete resection was documented while in another five patients, a second tumor surgery was mentioned. The NF-1 ratio in our cohort was relatively small (12.7%) compared to the HIT 1996 study cohort (27.3%) since children were only included when surgical therapy was applied during the course of treatment, which is less often indicated in NF-1.

The surgical approach of the HIT 1996 and HIT SIOP 2004 study was quite different from our recent strategy proposal [2, 10, 16]. Firstly, complete resection would never be the goal of surgery in CHG; secondly, repeated surgeries should be more liberally suggested as possible treatment strategies in balanced decisions towards other non-surgical treatment options. A similar approach was recently described technically by Kim et al., however, without offering outcome data to their cohort [17]. This is in line with Goodden et al., who proposed to reintegrate surgical strategies into the multimodal management of CHG [11]. In their cohort, 21 patients were treated surgically of whom 10 received surgery alone and survived with SD, and 11 patients received surgery together with chemotherapy of whom 10 remained in SD status during a median follow-up time of 77 months. One patient received an additional surgery. This cohort presents a smaller group of patients and a shorter follow-up time. Nevertheless, surgery was confirmed a relevant part of therapy in CHG. More modern surgical techniques such as pure endoscopic debulking techniques as also utilized in our cohort may further reduce the invasiveness in future approaches [12].

Repeated surgery in pLGG has been recently reported in a huge cohort of patients from the SIOP LGG 2004 study [16]. From 1271 surgically treated patients, 25.5% received at least a second surgery, while this proportion was higher in supratentorial midline tumor location (29.8%) that included CHG. This was relevant for the relative number of possible subsequent surgeries (up to 6 surgeries/patient) with a median follow-up time of 9.2 years. Thus, the acceptance of repeated but well-calculated surgeries in long-term management has increased.

Surgical complications in supratentorial midline low-grade glioma were more often seen after resection surgeries when compared to biopsy interventions [18]. In resection surgery, 41.2% experienced any post-operative neurological deficit in comparison to 15.3% after biopsy. The infection rate was relatively low (5.4 vs. 2.5%), and hemorrhages were seen in 15 vs. 4%, but none of the patients needed additional surgery. Seizures were seen in 8.1 vs. 2.5% which may be linked to the direct approach–related brain irritation or more likely to electrolyte concentration shifts due to pituitary hypothalamic manipulation. No data are available on long-term antiseizure medication. It was striking in the above mentioned study that complication rates were lower in big-volume centers when compared to high-volume centers, emphasizing the relevance of pediatric neurosurgical experience.

None of the studies so far included possible surgical interventions for tumor cysts or the total number of surgeries for CSF circulation disturbance. Goodden et al. reported a shunt rate of 52.4%, which is higher compared to our cohort with a shunt rate of 43%. Among our cohort, the shunt rate also differed between the two centers (Berlin, 39.5% vs. Tübingen, 50%). This may be explained by the amount of endoscopic interventions (3 stented ETV) being performed in Berlin using a stent placement for establishing the communication between internal and external CSF spaces [15]. However, in the Tübingen cohort, a significant part of children came to surgery from external units already with a shunt in place. On the other hand, the indication for tumor debulking inside the third ventricle to free the CSF spaces was one of the goals for surgical interventions to specifically avoid the implantation of a VP shunt.

Non-surgical treatment is key in LGG patients who experience continuous progressions and are mostly incurable by surgery which is often the case in CHG. In the HIT 1996 study, 63.5% of CHG were treated with chemotherapy and 16.6% with radiation [2]. In a more detailed analysis, 123 of 198 patients received vincristine/carboplatin treatment with a response rate of 92%. After a median follow-up time of 44.1 months, 44 patients still suffered from progression with the need for additional therapy [10]. That portion was generally similar in the SIOP LGG 2004 study with a portion of 70.9% (234/330 optic pathway glioma) receiving non-surgical treatment, of those, 44.5% received one non-surgical treatment, 11.8% received two, and 14.5% received three or more non-surgical treatment lines, each line yielding a lower PFS when compared to the first chemotherapy approach [19]. Systemic treatment in LGG patients is usually for 18 months which is a significant burden for patients and families. Likewise, radiation therapy is complicated by a high rate of neurocognitive impairment especially in younger age. Functional deficits are also observed after surgery. The risks and efficacy of each intervention should be wisely balanced to avoid long-term neurological sequelae. This is even more so in patients who need multiple treatment lines as each therapy has its own risks and benefits.

In recent years, targeted therapies for pLGG have become available [7–9] which are beyond the focus of this paper. Certainly, efficacy reports on MAPK inhibitors open new perspectives on tumor volume reduction [20, 21]. However, treatment-related adverse events were reported in a relevant portion of patients (42% ≥ grade 3), and 18.2% discontinued treatment due to adverse events or any kind of progression. Yet, it is a matter of debate, whether these therapies need to be given continuously. This adds another reason why it is important that the MDT decisions include all disciplines to decide about indication of novel drug treatment options. The number of targeted therapies applied in the current cohort was limited, and future experience will define the role of each therapy in the framework of CHG management.

In terms of outcome measures, we were able to show that three-quarters of patients reached SD, and 84% of patients were independently measured by the Karnofsky performance score. Visual acuity was preserved in 58% of patients with complete amaurosis in 16.2% of patients. Hormone substitution of any kind was seen in 30%, and 40% of children were overweight/obese. These outcomes results underline the challenge of the chronic condition of CHG on the one hand, but also open a perspective that these children participate in age-adequate daily activities. Our data are difficult to compare since most studies do not give details on outcome measures, which is difficult to retrieve after long periods of follow-up, or since all OPG patients are included also those who were not treated surgically [22–24]. Our results seem comparable to the Liverpool cohort, in which 39% of patients showed any kind of hypothalamic-pituitary dysfunction, and 74% were reported to have stable vision.

### Limitations

This study is limited by a first-glance relatively low number of 63 cases and its retrospective nature. On the other hand, the two center cohort offers a decent surgical experience for patients with CHG with a relatively long follow-up and will add to the existing literature on the topic. More accurate objective measurements on surgical resection such as volume changes after debulking and more detailed imaging data analysis postoperatively would be preferable. Similarly, outcomes in future studies will be more detailed. Instead of reporting detailed surgical complications, we therefore focused in this report on long-term outcome which presents the combined long-term consequences of the natural course of the disease and all treatments applied. Furthermore, we were able to give detailed information on tumor surgical interventions and also on hydrocephalus and cyst management, which are relevant cofounders to disease and outcome in this group of patients.

## Conclusions

**Fig. 3:**
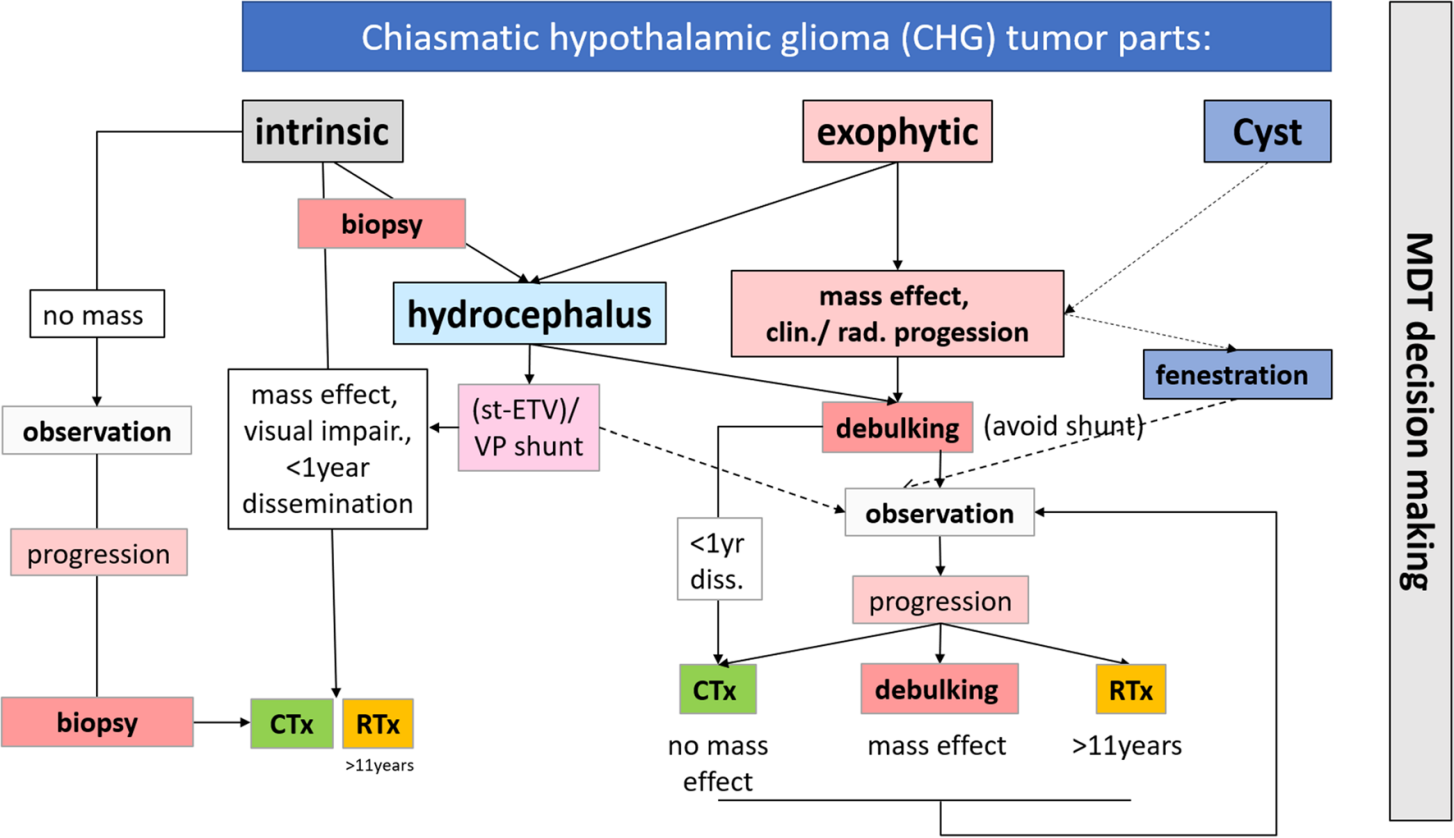
Schematic multidisciplinary treatment flow chart with emphasis on surgical options in the treatment of CHG. Exophytic tumor parts or cysts with clear mass effect or continuous progression, which are safely accessible represent indications for surgery. Tumor debulking is usually performed by trans-callosal approach or sub-frontally depending on the growth patterns of the tumor. Cyst fenestration can be achieved by navigated neuroendoscopy or by stent placement. Hydrocephalus treatment is achieved by tumor debulking to free the tumor from CSF pathways or if not feasible to perform stented ETV or VP shunt implantation. Biopsy is done in intrinsic tumor parts before systemic treatment preferably by neuroendoscopy in most cases. Observation or non-surgical treatment options depend on age, size of the lesion, possible dissemination, and dynamics of visual impairment. Radiation therapy is preserved for older children. In case of tumor progression after longer observation periods, new decisions are made on further treatment. All decisions should be made in the context of multidisciplinary case discussions that involve all expertise for the available treatment options

With the present study, we were able to show that surgical therapy plays a relevant role in the multidisciplinary care of the chronic and challenging condition of pediatric CHG. Not only biopsies and hydrocephalus management are relevant but also debulking surgeries of exophytic tumor parts which are safely accessible (typically T2 hyperintense signal tumor parts in MRI) carry benefits for the patient. Similarly, cyst fenestrations should be addressed by surgery, which can often be achieved by minimal invasive techniques such as navigated neuroendoscopy. In the framework of an interdisciplinary approach to these children, we basically distinguish between intrinsic, exophytic, and cystic tumor parts. The latter two conditions represent indications for surgery if tumor mass effects are relevant and/or progressive in nature and the tumor target zone is safely accessible. Hydrocephalus can be treated by tumor debulking of the third ventricular tumor part, stented ETV in a few cases, or VP shunt. These strategies are well embedded in the multidisciplinary decision-making framework of an experienced pediatric neurooncological center (Fig. 3). Future multicenter studies need to address in detail the question of surgical strategies and the extent of resection in relation to their efficacy of tumor disease control and safety for the patient.

## Data Availability

No datasets were generated or analyzed during the current study.
